# An application of ordinal regression to extract social dysfunction levels through behavioral problems

**DOI:** 10.3934/publichealth.2023041

**Published:** 2023-07-14

**Authors:** Alka Sabharwal, Babita Goyal, Lalit Mohan Joshi

**Affiliations:** 1 Department of Statistics, Kirori Mal College, University of Delhi, Delhi–91–110007, India; 2 Department of Statistics, Ramjas College, University of Delhi, Delhi–91–110007, India; 3 Department of Statistics, University of Delhi, Delhi–91–110007, India

**Keywords:** behavioral problems, negative log-log link function, ordinal regression, Strength and Difficulties Questionnaire, social dysfunction

## Abstract

Psychological problems are complex in nature and accurate identification of these problems is important. For the identification of psychological problems, one of the preliminary tools is the use of interviews/questionnaires. Questionnaires are preferred over interviews if the group under study is large. A strengths and difficulties questionnaire (SDQ) is one of the most widely used and powerful questionnaires to identify behavioral problems and distresses being faced by the respondents, affecting their day-to-day lives (responsible for social dysfunction). This study was held on college/university students in India, with the objective of examining if the extent of social dysfunction as measured by an impact score can be extracted from behavioral problems which are the components of the difficulty score of SDQ. Two surveys were conducted during the COVID-19 pandemic period, between the months of May–June 2020 and October 2020–February 2021 for the study. Only those responses were considered who felt distressed (“yes” to item 26 of SDQ). The numbers of such responses were 772/1020 and 584/743, respectively, in the two surveys. Distress levels were treated as ordered variables and three categories of distress level, viz., “Normal”, “Borderline”, and “Abnormal” were estimated through behavioral problems using ordinal regression (OR) methods with a negative log-log link function. The fitting of OR models was tested and accepted using Cox and Snell, Nagelkerke, and McFadden test. Hyperactivity-inattention and emotional symptoms were significant contributors to estimating levels of distress among respondents in survey 1 (*p* < 0.05). In addition to these components, in survey 2, peer problems were also significant. OR models were good at estimating the extreme categories; however, the “Borderline” category was not estimated well. One of the reasons was the use of qualitative and complex data with the least wide “Borderline” category, both for the “Difficulty” and the “Impact” scores.

## 1. Introduction

It is an established fact that the COVID-19 pandemic caused by the novel coronavirus (SARS-CoV-2) has affected people's mental health and behavior worldwide [1],[2]. Furthermore, preventive measures such as isolation and quarantine aggravated the problem and people experienced significant levels of anxiety, anger, confusion, and stress [3]. One of the most affected groups due to the pandemic and its consequences was the young adults enrolled in higher education, as they were exposed to an additional consequence of uncertainty regarding academic success, future careers, and social life during college, among other concerns [4]. The psychological health issues of this group have become a primary concern of psychological health practitioners and researchers across world.

Psychological problems may be very complex in nature and may have long-lasting effects. As such, the clear and appropriate identification of these problems is very important to deal with these problems. The choice of an appropriate tool is the first step toward the identification of the problem. One of the standard tools used by researchers is a questionnaire, which has been designed for a specific method and targets a specific group [5]. For example, the Patients Health Questionnaire (PHQ-9) is a 9-item questionnaire and is widely used to measure the severity of depression [6]. The Generalized Anxiety Disorder Scale-7 (GAD-7) is a 7-item, self-rated screening tool used for generalized anxiety disorders [7]. These tools can be administered to groups of respondents, as well as to an individual respondent.

The Strengths and Difficulties Questionnaire (SDQ) is a brief instrument used to measure psychological behavior problems and social dysfunction of a respondent and assesses both strengths and difficulties simultaneously [8],[9]. There are many versions of SDQ which have been designed according to the needs of different target groups. The 4–11 years SDQ version is for the parent/teacher of the subject. The 11–17 years version is used by the subject as well as their parent/teacher. The 17^+^ version, which has been used in this study, is a self-assessment questionnaire. Currently, there are three versions of the SDQ for each of these age groups: a short/basic version with 25 items, a longer form/extended version with an impact supplement, and an extended version with an added follow-up form. The 25 items of the basic version of the questionnaire are further categorized into five scales: the first scale (prosocial behavior) is the strength scale; and the remaining four scales are difficulty scales (namely, “conduct problems”, “peer problems”, “emotional symptoms”, and “hyperactivity-inattention”). The extended versions of the SDQ further enquire about chronicity, distress, social impairment, and burden to others through items 28–33. These five items, along with item 27 are answered only if the response to item 26 is “yes” (i.e., if the respondent feels difficulties in areas of emotions, concentration, behavior or being able to get along with other people). Item 27 measures the duration of distress and item 33 measures the burden of distress on the family and friends of the respondents.

A useful analysis of psychological data involves the identification and execution of an appropriate statistical technique. The psychological data is generally categorical in nature and many quality-of-life scales are ordinal. In order to estimate categorical response variables through independent predictors, in earlier works, ordinal and multinomial regression models have been found quite useful. Previous works have suggested that the classification for medical diagnosis is ordered, which corresponds to the level of health risk. Ordinal regression (OR) models provide an appropriate strategy for analysing the effects of multiple explanatory variables on an ordered, observed categorical outcome that cannot be assumed to be a continuous measurement with normal distribution [10]. In OR analysis, link functions are used to build specific models. Some of the commonly used link functions are logit, complementary log-log, negative log-log, probit, and Cauchit link functions, which are chosen on the basis of the characteristics of the underlying data. Generally, the logit link is considered suitable for analysing ordered categorical data evenly distributed among all categories; the complementary log-log link is often used when higher categories are more probable, whereas with a negative log-log link function, lower categories are more probable [11].

The OR models have been frequently used in medical data. A vast literature is found on applications of OR models and their variants used in medical and bio-statistical data. The proportional odds and partial proportional odds models have been used by the following: by Lall et al. (2002) to study cognitive function health and aging [12]; by Liu et al. (2018) in Diabetic Retinopathy Diagnosis (DR) with five risk levels [13] and in Breast imaging reporting cancer [14]; by French & Shotwell (2022) assessed COVID-19 status 14 days after a randomization test on a seven point scale, [15]; and by Wolde et al. (2022) to study three levels of hypertension [16], to name a few.

In this study, the OR has been used to estimate the categories of distress resulting in social dysfunction using the impact scores of SDQ. Using the SDQ 17^+^ extended version, two surveys were conducted during the COVID-19 pandemic: the first during the months of May–June 2020; and the second during the months of October 2020–February 2021. The aim of the surveys was to assess the impact of COVID-19 on the mental health of 18–25 years old college/ university students. The numbers of responses in the two surveys were 1,020 and 743, respectively. The data reliability was tested using Cronbach alpha and Guttman Lambda. The questionnaire had two components, namely “Difficulty” and “Impact” scores of SDQ, to measure behavioral problems and social dysfunction respectively. Furthermore, a study was conducted to understand if the two scores provide similar conclusions about the mental health of the respondents; under the hypothesis that the impact scores in “Normal”, “Borderline”, and “Abnormal”, bands can be estimated with “Difficulty” scores in the same bands. A hypothesis was tested by formulation of the ordinal models to estimate the probability/category of impact scores with independent predictors; conduct problem, peer problem, emotional symptoms and hyperactivity-inattention for every participant using a negative log-log link function of the form −ln(−ln(*F_k_*(*x_i_*))) was tested by applying Cox and Snell, Nagelkerke, and McFadden test statistics to the model. The significance of the predictor was obtained using Wald statistics. Significant factors obtained for each category were compared to the base stage and the cutoff points. Using the fitted model, the category of distress of each respondent was predicted. The assumption of parallel lines was tested since the odds ratio was same for different categories of distress. Finally, a comparison between the predicted category and the observed category was obtained.

The novelty of the study was that the population under investigation was not unhealthy. These were psychologically healthy individuals but were facing unprecedented, unhealthy times. The study collected the data for the same population twice, at a gap of one year, when the levels of severity of the effect of the pandemic were not the same in the Indian subcontinent. The study clearly indicated the effect of the pandemic on the psychological health of the respondents; additionally, it estimated the predictive efficiency of the behavioral scales on the social dysfunction of these respondents during pandemic times. To the best of our knowledge this is the first study of its kind in India involving statistical modeling based on two surveys conducted during pandemic times on the same population throughout the country. Besides the introduction, the course of the paper is as follows: material and methods are explained in Section 2; results are discussed in Section 3, which are followed by a discussion in Section 4 and a conclusion in Section 5.

## 2. Materials and methods

### 2.1. Material

During the COVID-19 pandemic period, data were collected through two surveys conducted in online and offline modes, on students studying in various colleges and higher educational institutes across India using the SDQ 17^+^ self-reported extended version. The surveys were conducted as follows: i) in the months of May–June 2020 almost two months after a nationwide lockdown was imposed; and ii) in the months of October 2020 to February 2021. The first survey was conducted entirely in the online mode and 1,020 students participated in the study. The survey gathered information on demographic variables such as age and gender, and 33 items of the SDQ 17^+^ questionnaire. The second survey was conducted both in online and physical modes and 743 undergraduate and postgraduate students participated in it. The questionnaire was divided into two sections. The first section had questions regarding the demographic details of the respondents such as their age, gender, place of living, family composition, and family income, along with details of the direct impact of COVID-19 in terms of the occurrence of the disease and resulting hospitalization in the family (including themselves) of the respondents. The second section (common in surveys 1 and 2) of the questionnaire was based on the SDQ 17^+^ extended version. The SDQ scores were categorized according to the standard classification of cut-off points in the SDQ manual [17].

### 2.2. Methods

OR models belong to the class of generalized linear regression models as they allow for a more generalized distribution of error terms that differs from the normal distribution of errors. OR models are used to predict ordinal-level dependent variables with a set of independent variables. The first category is usually considered the lowest category, the last category is the highest category (numerically coded from 0 on up), and the independent variable may be either categorical or continuous [18].

Let *y_i_* be the *i*^th^ individual response *i* = 1, 2 ... *n* and y_i_* be the corresponding latent variable. The OR model makes the assumption that y_i_* (and not *y_i_*) depends on *x_i_*, i.e.

$$y_{{}_{i}}^{*}=x_{i}^{\prime }\underset{˜}{\beta }+\varepsilon_{i};\;i=1,2...n,$$ (1)

where $\underset{˜}{\beta }$ is the vector of regression coefficients needed to be estimated and y_i_* is the unobserved dependent variable. The relationship between y_i_* and the observed variable *y* is as follows:

$$y=\left\{ \begin{array}{l} 1\text{ if }0\leq y^{*}\leq \theta_{1}\\ 2\text{ if }\theta_{1}\leq y^{*}\leq \theta_{2}\\ \vdots \\ N\text{ if }\theta_{N-1}\leq y^{*} \end{array}\right..$$ (2)

Let *p*_1_(*x_i_*), *p*_2_(*x_i_*), ..., *p_k_*(*x_i_*) denote the response probabilities at values for a set of explanatory variables. The cumulative probabilities are given by:

$$F_{k}\left(x_{i}\right)=P\left(Y\leq k|X_{i}\right)=p_{1}\left(x_{i}\right)+p_{2}\left(x_{i}\right)+...+p_{k}\left(x_{i}\right);\;k=1,2,...K-1.$$ (3)

Define

$$L_{k}\left(x_{i}\right)=\log it\left(F_{k}\left(x_{i}\right)\right);\;k=1,2,...K-1$$ (4)

where *F_k_*(*x_i_*) is the cumulative probability up to and including category *K*.

Then the proportional odds model [19] is given by

$$L_{k}\left(x_{i}\right)=\alpha_{k}+\beta_{k}^{\prime }x_{i};\;k=1,2,...K-1.$$ (5)

The parameters *α*_1_, *α*_2_, ..., *α_k_*_−1_, are non- decreasing in *k* and are known as the intercepts or the “cut-points”. The parameter vector $\underset{˜}{\beta }$ contains the regression coefficients for the covariate vector ${\underset{˜}{x}}_{i}$. Inherent in this model is the proportional odds assumption, which states that the cumulative odds ratio for any two values of the covariates is constant across response categories or the “parallel line assumption”, which states that there is one regression equation for each category except the last category. The last category probability can be predicted as the second last category probability.

The model contains the *K*-1 response curves of the same shape, and therefore we cannot fit it by fitting separate *logit* models for each cut-point. Then, we maximize the multinomial likelihood, subject to constraints. The model assumes that the effects of the variables are the same for each cut-point, *k* = 1, 2... *K*−1.

One advantage of an ordered analysis over the corresponding nominal analysis is that, generally, fewer parameters are needed to describe a model for the response [20]. As a result, the ordinal regression models are more powerful.

In order to fit generalized linear models to ordinal response outcomes, distinct “link functions” are used to link the (cumulative) response to the set of predictor variables. Various available link functions used have been tabulated below in Table 1 [11].

**Table 1.** Various link functions used in Ordinal Regression methods.

Link function	Form	Conditions to be used
Logit	$\ln\left(\frac{F_{k}\left(x_{i}\right)}{1-F_{k}\left(x_{i}\right)}\right)$	Categorical data is evenly distributed among all categories. Here, the errors are distributed according to a logistic distribution.
Probit	Φ^−1^(*F_k_*(*x_i_*))	Probit regression assumes that the errors are distributed normally.
Complementary log-log	ln(−ln(1−*F_k_*(*x_i_*)))	For skewed data, when higher categories are more probable.
Negative log-log	−ln(−ln(*F_k_*(*x_i_*)))	For skewed data, when lower categories are more probable
Cauchit	tan(*π*(*F_k_*(*x_i_*)−0.5))	This type of link bears the same relation to the Cauchy distribution as the probit link bears to the normal. One characteristic of this link function is that the tail is heavier relative to the other links.

Norusis (2012) [21] suggests the choice link function should be based on the distribution of the response variable. In this study, we have used a negative log-log link function [22].

#### 2.2.1. Parallel lines assumption

In OR models, there is an important assumption which states that the correlation between the independent variable and dependent variable does not change for the dependent variable's categories; additionally, parameter estimations do not change for cut-points. In other words, this assumption states that the dependent variable's categories are parallel to each other. The likelihood ratio test, Wald Chi-Square test, and other related tests are used to test parallel lines assumption [23],[24]. In OR, these tests examine the equality of the different categories and decide whether the assumption holds. If the assumption does not hold, interpretations about results will be wrong; therefore, in order to find correct results, alternative models are used instead of the ordinal logit regression models. The hypothesis that tests whether coefficients *β_k_* of independent variables are equal or not is tested for every single category.

$$H_{0}:\beta_{1j}=\beta_{2j}=...=\beta_{\left(k-1\right)j}=\beta_{j};j=1,2,...J$$ (6)

### 2.3. The goodness-of-fit tests

The null hypothesis for the goodness-of-fit tests is that the model fits the data well against the alternative hypothesis, which refers to an unspecific problem with the fit. Thus, a small *p*-value is an indication of lack of fit of the model. The following are the three pseudo-*R*^2^ statistics for OR.

**Table 2.** Test statistics for testing the goodness of fit of an ordinal model.

Test	Formula	Explanation
McFadden's *R*^2^	$R_{L}^{2}=1-\frac{LL_{\mathrm{mod}el}}{LL_{0}}$	This is the natural logarithmic linear ratio *R*^2^. A value close to 0 indicates that model has no predictive value
Cox and Snell's *R*^2^	$R_{CS}^{2}=1-{\left(\frac{LL_{0}}{LL_{\mathrm{mod}el}}\right)}^{\frac{2}{n}}$ *n*= sample size	This is a “generalized” *R*^2^ (used in linear regression as well) rather than a pseudo *R*^2^. A problem with this *R*^2^ is that the upper bound of this statistic, given by $1-{\left(p^{p}{\left(1-p\right)}^{1-p}\right)}^{2}$ is less than 1where *p* is the marginal proportion of cases with events.
Nagelkerke's *R*^2^	$R_{Nagel}^{2}=\frac{R_{CS}^{2}}{1-e^{\frac{2LL_{0}}{n}}}$	It measures the proportion of the total variation of the dependent variable can be explained by independent variables.

where

*LL_model_* = full log-likelihood model including all coefficients (depending on the number of predictors);

*LL_0_* = log-likelihood model with fewer coefficients (model with only the intercept *b*_0_); ln(*L*_0_) being analogous to residual sum of squares in linear regression.

## 3. Results

In order to study the effect of COVID-19 on the psychological health of college/university students, two surveys were conducted in online and offline modes using the SDQ 17^+^ extended version. Approximately 1,020 and 743 students participated in survey 1 & survey 2, respectively. Among these, 462 (45.29%), and 383 (51.55%) were males in survey 1 and survey 2, and 558 (54.71%) and 360 (48.45%) females, respectively. The participants were from across several streams viz. humanities, commerce, sciences, law, management, engineering, medicine, nursing, and interns. All the responses were scored according to the SDQ manual. All five scales of the SDQ manual for all the participants were valid scores in both surveys. Table 3 below presents the descriptive statistics of all the items of SDQ; first the five scales of five items each and “Impact” scores for only those respondents who answered yes to item no 26 students under both the surveys stratified gender-wise.

**Figure 1.**
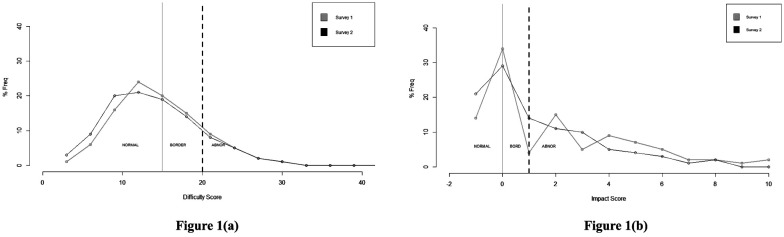
Categories of difficulty (a) and impact scores (b) as identified by the cut-off points.

The SDQ was designed to screen for behavioral problems in youths based on cutoff points that favor the instrument's diagnostic sensitivity [9],[16]. Graphically, we have displayed the cutoff points of three SDQ categories of all the respondents who participated in both surveys. Figure 1a presents the “Normal”, “Borderline”, and “Abnormal” categories, defined by the cutoff points of the “Difficulty” score in two surveys. It can be observed from Figure 1a that students with lower scores have a higher frequency than students with higher scores. However, there are more than 30% of respondents are in the affected groups (facing behavioral problems). Figure 1b depicts the proportion of respondents with “Impact” score of two surveys in different categories viz. “No distress”, “Normal”, “Borderline”, and “Abnormal”. It can be observed that students with a score < 1 are in the Normal band (either the answer to item no 26 is “no” or the impact score is 0) and there are more than 45% are in the affected groups (i.e., facing social dysfunction during the surveys).

**Table 3.** Descriptive statistics of two surveys giving mean, standard deviation, median, mode, minimum, and maximum of five strength and difficulty scales; and Difficulty and Impact scores.

Scale(Items)		Total	Mean	Sd	Minimum	Maximum
Prosocial behaviour (1, 4, 9, 17, 20)	Survey 1	772	7.891	1.686	1	10
	Survey2	584	7.932	1.754	0	10
Hyperactivity-inattention (2, 10, 15, 21, 25)	Survey 1	772	4.104	2.034	0	9
	Survey2	584	3.724	2.059	1	10
Emotional symptoms (3, 8, 13, 16, 24)	Survey 1	772	4.193	2.450	1	10
	Survey2	584	3.995	2.488	0	10
Conduct problem (5, 7, 12, 18, 22)	Survey 1	772	2.935	1.456	0	9
	Survey2	584	2.785	1.441	1	7
Peer problem (6, 11, 14, 19, 23)	Survey 1	772	2.902	1.713	0	10
	Survey2	584	2.942	1.787	0	9
Difficulty score	Survey 1	772	14.136	5.142	1	31
	Survey2	584	13.443	5.568	2	33
Impact Score (28, 29, 30, 31, 32)	Survey 1	772	1.528	1.721	0	7
	Survey2	584	1.885	2.100	0	9

### 3.1. The probability/category of impact Score of every respondent with the ordinal regression model

Ordinal models have been applied to estimate the probability/category of the impact score with the following independent predictors for every participant: conduct problem, peer problem, emotional symptoms, and hyperactivity-inattention. The difficulty scores of those respondents have been considered whose impact scores are available. The data (Figure 1a,b) suggest that the lower values of the impact score have a higher frequency than the higher values. Thus, the negative log-log link function is most appropriate for the OR model to be used.

As a first step of OR analysis, the intercept model is compared with the full model. Null hypothesis and alternative are:

*H*_0_: Intercept model is good;

*H*_1_: Full model is good.

The results are presented in Table 4 below.

**Table 4.** Comparison of Full model with intercept model.

	Model	-2 Log Likelihood	Chi-Square	Df	Significance
Survey_1	Intercept Only	1590.920			
	Full	1486.807	144.113	4	<0.001
Survey_2	Intercept Only	1186.088			
	Full	1031.238	154.851	4	<0.001

The full model was found to be good for both surveys with a *p*-value < 0.001. Furthermore, the fitting of OR models with the negative log-log link function is tested using Cox and Snell, Nagelkerke, and McFadden test. The models were found to be appropriate for both surveys with *p*-values > 0.05. The upper bound of Cox and Snell *R*^2^ was found to be 0.952248 for *p* = 0.44 in survey 1 and 0.729325 for *p* = 0.36 in survey 2.

### 3.2. Social Dysfunction estimated with behavioral problems using Ordinal Regression

In the present study, by applying OR, the interest lies in deciding whether or not the predictors have the predictive efficiency of the model. The values of the regression coefficients for hyperactivity-inattention, conduct problems, emotional symptoms, and peer problems factors account for the size of the effect that a variable is having on the dependent variable, and the sign of the coefficient gives the direction of the effect. It has been found that hyperactivity-inattention and emotional symptoms are significant contributors for estimating respondents' probability of belonging to a category, as *p* < 0.05 in survey 1. It has been found that peer, hyperactivity, and emotional are significant contributors for estimating respondents' probability of belonging to a category, as *p* < 0.05 in survey 2. The conduct problem is not a significant factor in both surveys. The detailed results are given below in Table 5. If the response variable takes the value 0, it means that the respondent is under the normal category (distress is not affecting social dysfunction); if the response variable takes the value 1 / value 2, it means that the respondent is under the Borderline / Abnormal category (presence of social dysfunction). The detailed results are given below in Table 5.

**Table 5.** Ordinal Regression showing the partial effects of components of difficulty scales on impact scores of the participants in the two surveys.

Survey 1

		Estimate	Std. Error	Wald	Df	Sig.	95% Confidence Interval	
							Lower Bound	Upper Bound
Threshold	[impact = 0]	1.217	0.148	67.784	1	<0.001	0.927	1.506
	[impact = 1]	1.810	0.155	136.651	1	<0.001	1.506	2.113
Location	Hyperactivity	0.096	0.025	15.113	1	<0.001	0.048	0.144
	Emotional	0.176	0.022	66.691	1	<0.001	0.134	0.219
	Conduct	-0.030	0.032	0.894	1	0.344	-0.094	0.033
	Peer	0.023	0.028	0.655	1	0.418	-0.032	0.077

Survey 2

Threshold	[impact = 0]	1.387	0.161	74.722	1	<0.001	1.073	1.702
	[impact = 1]	2.117	0.174	148.813	1	<0.001	1.777	2.457
Location	Hyperactivity	0.110	0.030	13.817	1	<0.001	0.052	0.168
	Emotional	0.197	0.026	58.552	1	<0.001	0.147	0.248
	Conduct	-0.038	0.036	1.081	1	0.298	-0.034	0.109
	Peer	0.063	0.031	4.021	1	0.045	0.001	0.124

**Estimated OR model for Survey 1**

$Y_{Nor-Bor}=1.217-0.096HA-0.176EC-\left(-0.030CP\right)-0.023PC$

$Y_{Bor-Ab}=1.810-0.096HA-0.176EC-\left(-0.030CP\right)-0.023PC$

**Estimated OR model for Survey 2**

$Y_{Nor-Bor}=1.387-0.11HA-0.197EC-\left(-0.038CP\right)-0.063PC$

$Y_{Bor-Ab}=2.117-0.11HA-0.197EC-\left(-0.038CP\right)-0.063PC$

where

$$Y_{Nor-Bor}=-\log\left(-\log\left(\frac{P\left(participants\;are\;in\;Normal\;category\right)}{P\left(participants\;are\;in\;Borderline\;category\right)}\right)\right)$$

$$Y_{Bor-Ab}=-\log\left(-\log\left(\frac{P\left(participants\;are\;in\;Borderline\;category\right)}{P\left(participants\;are\;in\;Abnormal\;category\right)}\right)\right)$$

*HA*≡ Hyperactivity-inattention score

*EC*≡ Emotional symptoms score

*CP*≡ Conduct problem score

*PC*≡ Peer problem score

OR models are based on the assumption of parallel lines (i.e., parameter estimations do not change for cut points). In other words, the dependent variable's categories are parallel. The assumption is needed for an accurate interpretation of the results. To test this assumption, the following null and alternative hypotheses were set:

*H*_0_: The slope coefficients of predictors in the model are the same across all response categories.

*H*_1_: The slope coefficients of predictors in the model are not the same at least for one of the response categories.

The significance values are found to be 0.071 and 0.251 for surveys 1 and 2, respectively. The proportional odds/parallel lines assumption is accepted. The detailed results are presented in Table 6 below.

**Table 6.** Test of parallel lines.

	Model	-2 Log Likelihood	Chi-Square	Df	Sig.
Survey 1	Null Hypothesis	1446.807			
	General	1438.185	8.622	4	0.071
Survey 2	Null Hypothesis	1031.238			
	General	1025.865	5.372	4	0.251

### 3.3. Comparison of the estimated categories with the observed ones

The principal objective of the study is to estimate impact scores with behavioral problem (difficulty) scores. For this, the probability of each category of impact score (indicating social dysfunction) through behavioral problems has been computed for all the respondents. The criterion for categorization of distress is that the probability of that category should be highest among all the categories. The comparison between the observed and estimated bands of impact scores for both surveys is presented in Table 7 below.

The OR model estimated the observed normal band as the normal category and the observed advance band as the advance category, with almost 70% accuracy. The model has good predictive power, but it fails to estimate slightly raised (Borderline) band under all the categories, despite the model being an appropriate one in terms of prerequisites as enlisted in Table 1. Furthermore, it is clear from the estimated results that there were young adults (16.5% in survey 1 and 30.5% in survey 2) whose difficulty score was under the normal band, but they still faced the advance level of social dysfunction. This means that for these participants, the difficulty scores were less than 15; however, they were facing “a great deal” problem under at least one area of behavior problems resulting in an abnormal level of distress causing social dysfunction. On the other hand, if respondents were under the advance category of behavioral problems, then almost everyone experienced distress (more than 90% in survey 1 and 99% in survey 2). All the analysis has been done in SPSS, version 26 and R software, version 4.2.1.

**Table 7.** Comparison between the observed and estimated bands of impact scores for both the surveys.

Survey 1						

		Impact Score				Total
			No problem	Slightly raised	Advanced	
Difficulty Score	Observed	No problem	283	34	172	489
		Slightly raised	46	28	94	168
		Advanced	14	24	77	115
	Estimated	No problem	408	00	81	489
		Slightly raised	34	00	134	168
		Advanced	02	00	113	115

Survey 2						

Difficulty Score	Observed	No problem	190	73	128	391
		Slightly raised	19	21	66	106
		Advanced	05	09	73	87
	Estimated	No problem	272	00	119	391
		Slightly raised	00	00	106	106
		Advanced	00	00	87	87

## 4. Discussion

The subjects of this study were young adults who were otherwise psychologically healthy; however, they were facing unprecedented problematic trials during the COVID-19 pandemic period. They were investigated to determine the effect of the pandemic on their psychological health. The SDQ (extended version) was chosen for the purpose for data collection due to its effectiveness and reliability in studying behavioral problems, as well as social dysfunction in a generally healthy population of young adults.

In order to study a statistical relationship between the categories of two components of SDQ scores, namely, the difficulty and the impact scores, the OR model was selected because it is a robust technique; in case the response variable is an ordered variable with few categories and mutually exclusive categories, these can be ordered by their clinical preference. This model has been used repeatedly in medical and bio statistical studies and has been useful in estimating the output variable (stages of disease) in diseases like cancer and chronic kidney disease with independent predictors. The models have been applied in COVID-19 related studies with as aims such as the identification of factors responsible for COVID-19 infection by application of a geographically weighted ordinal logistic regression model and the effect of space over these factors [25], and the effect of various treatments for the disease by assessing COVID-19 status 14 days after a randomization test on a seven point scale [15].

The choice of an appropriate link function is of crucial importance in OR. As the numbers of respondents in normal categories were highest for both the social dysfunction as well as behavioral problems, the most suitable link function is the negative log-log link function, as it is used when the lower values are more probable. The statistical relationship was examined by obtaining the following predictive efficiency of predictors: hyperactivity-inattention, conduct problems, peer problem, and emotional symptoms scores about the level of distress causing social dysfunction. One of the strengths of OR models is that OR considers the items and participants, incorporating all data information into the model, and controls for dependencies between ratings from the same person and between ratings of the same item. The parameters are the multiple intercepts that are thresholds/ cut points.

The cut points in the data of the present study indicate the levels of distress of the respondents. About 70% of respondents' category of distress is correctly estimated by the applied model. The predictive efficiency of the model was quite good. However, respondents who observed a “Borderline” difficulty score either were either under or over-estimated by this model. This is due to the complex and multi-component data collected through a SDQ questionnaire, which not only is subjective in nature but takes values in limited and narrow categories. For an impact score to lie in the “Abnormal” band, either the respondent has at least two or more problem areas in “quite a lot” category or at least one problem area in the “a great deal” category, while some other areas may be in the “not at all” or “only a little” categories. For a score to lie in the “borderline” band, the respondent has at most one problem area in “quite a lot” category and “no problem” in other areas. However, for the independent predictors, they contribute to the difficulty score of the respondent, indicating the behavioral problem. For a score to lie in the “borderline” band, the respondent has to have two/three problem areas in “quite a lot” category, or two problem areas, out of which one is in the “quite a lot” category and one is in the “the great deal” category. As an example, on the basis of scores of three respondents in survey 1, which are (2,5,7,4), (3,6,6,4), and (3,5,5,5), all the three are in the borderline category of the difficulty score (as per total). However, the first respondent has a “quite a lot” problem in one area (conduct problem) and “a great deal” problem in one area (peer problem); the second respondent has “quite a lot” problems in two areas (conduct problem and peer problem) and the third respondent has “quite a lot” problems in three areas (conduct problem, peer problem and emotional symptoms). The complexity of the data is evident from the fact that a respondent with a “normal” category difficulty score had the scores in individual scales of 1,2,2,8. Emotional conduct of this respondent was in “a great deal” band.

For both the surveys, the OR model estimated the impact scores of all the respondents having the “borderline” difficulty scores; however, with two problem areas, one in “a great deal” category while the other in “quite a lot” category or with three problem areas, all in the “borderline” category or in the “abnormal” category. This means that all those cases that were in the “borderline” category as per difficulty score but estimated as “abnormal” category of impact score might be as problematic as the “abnormal” difficulty score cases. While the OR model provides a reasonably good relationship between extremes category case (i.e., “normal” and “abnormal” difficulty and impact scores), it also suggests the case-by-case investigation of “borderline” cases. Therefore, the OR model has been able to provide useful additional information for clinicians and researchers with an interest in psychiatric scores. All the “borderline” cases and the “normal” cases with scores close to being “borderline” should be investigated further to determine the need of expert intervention.

The results of the study are consistent with earlier studies. The observed and the empirical conclusion that up to 50% of the respondents (both males and females) were facing severe distress corroborated the findings of the earlier studies, which suggested that a very high proportion of young adults were facing severe mental health issues during the pandemic times [26],[27].

The novelty of this study is the assessment of the general psychological behaviour of a healthy population in unhealthy times, not only through observations but also through statistical modelling. The study clearly shows the deviations of the population proportions from standard population proportions of (normal: borderline: abnormal) 80%:10%:10% in normal times. Additionally, the study emphasizes the need of case-by-case investigation of ‘borderline’ and ‘close to borderline’ cases if the questionnaire has been administered to a group of young adults.

The SDQ 17^+^ version is meant to identify the psychological problems of young adults. However, the data was collected mostly online from the young adults enrolled in higher educational institutions, thus limiting the scope of investigation to such young adults only in this study. Further the investigators were not in direct contact with the respondents at the time of data collection and therefore could not ensure the requirements of answering the SDQ (i.e., following time limit and not revisiting the responses). However, the data of the two surveys were consistent and had good reliability quotients. In the future, the model can be applied to a larger group of respondents, not necessarily students only. Additionally, the application of the model on the time series data may provide useful insight to the clinicians about the respondents' behaviour on a mass scale as well as for individual respondents.

## 5. Conclusions

OR models are good at estimating the extreme categories, though the “Borderline” category was not estimated well. One of the reasons was the use of qualitative data with the least wide “Borderline” category, both for the difficulty and the impact scores. Normal difficulty scores do not necessarily indicate the absence of distress but advance levels of difficulty scores correspond to advance levels of distress. Even normal difficulty scores can have components lying in “quiet a lot” of “a great deal” categories. Such cases should be dealt individually. Extended version of SDQ should be preferred over the commonly used basic version of the questionnaire.

## Use of AI tools declaration

The authors declare they have not used Artificial Intelligence (AI) tools in the creation of this article.
